# Weed Segmentation in Soybean Fields and Variable-Rate Herbicide Prescription Map Generation Based on UAV Imagery and Improved YOLOv11-seg Model

**DOI:** 10.3389/fpls.2025.1743263

**Published:** 2026-02-10

**Authors:** Yaohua Yue, Anbang Zhao

**Affiliations:** 1 College of Engineering, Heilongjiang Bayi Agricultural University, Daqing, China; 2 College of Information and Electrical Engineering, Heilongjiang Bayi Agricultural University, Daqing, China

**Keywords:** GIS, precision agriculture, soybean seedlings, UAV, weed segmentation, YOLOv11-seg

## Abstract

**Introduction:**

Weeds pose a major threat to soybean yield during the early seedling stage, where accurate identification of their spatial locations and contours is essential for precise field management. This study proposes an improved UAV-based YOLOv11-seg framework for high-precision weed segmentation in soybean fields.

**Methods:**

A real-field weed dataset was established under complex agricultural environments. A UAV-inspection-oriented, task-driven improved YOLOv11-seg weed segmentation method is proposed. The core of this method lies in the targeted integration and adaptation of existing modules to optimize small-target perception. To enhance detection accuracy, the backbone and neck C3K2 modules were replaced with RCSOSA (reparameterized convolution based on channel shuffle and one-shot aggregation). A Spatially Enhanced Attention Module (SEAM) was integrated into the C2PSA block to better distinguish small weeds from soybean seedlings, while the inverted Residual Mobile Block (iRMB) and adaptive down-sampling module (ADown) improved feature representation and reduced detail loss in low-contrast scenes.

**Results:**

Experimental results show that the proposed model achieves mAP@0.5(Box) = 0.89 and mAP@0.5(Mask) = 0.84, surpassing mainstream models such as YOLOv8s-seg and YOLOv12s-seg, with lower computational cost (25.3 GFLOPs, 8.3 M parameters).

**Discussion:**

The main contribution of this study lies in establishing a complete and practical end-to-end engineering workflow, spanning from accurate UAV image recognition to the generation of variable-rate application prescription maps. By integrating with the ArcGIS Pro platform, this solution achieves a fully automated pipeline from perception to decision-making, offering reliable technical support for intelligent weed control during the seedling stage in precision agriculture.

## 1 Introduction

Weeds in soybean fields during the seedling stage are a major biotic stress that significantly affects crop yield and quality. They compete with soybean plants for water, nutrients, and light, and may serve as intermediate hosts for pests and diseases, leading to physiological damage and ecological imbalance, ultimately causing substantial reductions in yield and deterioration of crop quality (Bali et al., 2016; Chetan et al., 2016). In northern China, the predominant soybean-producing regions primarily employ post-sowing pre-emergence closed weeding techniques, which exploit the germination time difference between crops and weeds for effective control. However, conventional large-scale uniform herbicide applications lack spatial specificity, often resulting in overuse of chemicals, environmental pollution, increased weed resistance, and degradation of agricultural ecosystems. With the ongoing transition toward sustainable and environmentally friendly agriculture, developing methods for seedling-stage weed detection and herbicide application map generation based on precise segmentation and recognition is crucial for achieving quantitative, site-specific, and timely herbicide application over large fields, with both theoretical and practical significance (Jingxu, 2023).

In computer vision and image processing, field weed recognition is essentially a multi-object segmentation and classification problem under complex environmental conditions. This task requires discriminating and extracting different target categories based on multidimensional image features, such as color, texture, shape, and spatial distribution, to semantically distinguish weed regions from crop regions (Hongbo and Nudong, 2020). Traditional image segmentation methods, which often rely on manually defined thresholds or handcrafted feature extraction algorithms, are susceptible to interference from illumination variations, soil background, occlusion, and overlap in natural field environments, resulting in unstable recognition performance (Bakhshipour and Jafari, 2018; Chunjian, 2022; Rehman et al., 2018). Recently, rapid advances in UAV-based low-altitude remote sensing have enabled researchers to acquire high-resolution canopy imagery over large farmland areas within a short period (Cui et al., 2024; Luo et al., 2025). Such data contain rich spectral and textural information and offer temporal and spatial controllability, providing a solid foundation for crop condition analysis and weed spatial distribution monitoring (Guo et al., 2025; Xu et al., 2025). However, UAV imagery is characterized by high dimensionality, complex backgrounds, and significant scale variation, which often limit the performance of traditional computer vision methods and necessitate more efficient deep learning models (Luo et al., 2025; Sheng et al., 2020; Shengsheng et al., 2019).

The introduction of deep learning has provided a novel avenue for weed recognition in agriculture. Compared with traditional machine learning methods that rely on handcrafted features, convolutional neural network (CNN)–based object detection models can automatically learn feature representations in an end-to-end manner. Among them, the YOLO (You Only Look Once) series of single-stage detectors have been widely adopted in crop object detection due to their fast detection speed and high computational efficiency (Lu et al., 2025; Sulzbach et al., 2025; Zhang et al., 2025; Zhao et al., 2025). Nevertheless, directly applying object detection to soybean seedling-stage weed recognition faces multiple challenges: (1) weeds and soybean seedlings are highly similar in morphology, color, and texture; (2) seedling-stage vegetation is densely distributed, exhibits diverse postures, and frequently overlaps; and (3) targets are small with indistinct boundaries, leading to overlapping detection boxes and localization errors. These factors constrain the effectiveness of bounding box–based detection methods for precision weeding tasks.

To overcome these challenges, instance segmentation techniques have been integrated into the YOLO framework (Gu et al., 2025; Guo et al., 2025; Zhong et al., 2025), enabling pixel-level recognition and contour extraction for each object in the image (Xu et al., 2025)addressed variable weather challenges in weed detection through CycleGAN-based domain adaptation and fine-grained segmentation with ConvNeXt, validated by its state-of-the-art performance in soybean fields (Genze et al., 2022) tackled UAV challenges (motion blur, occlusions) in sorghum weed detection via a generalized model and a dedicated dataset, enabling effective intra-row weed detection without auxiliary information and achieving an F1-score >89%.This approach not only provides spatial information of targets but also accurately delineates their boundaries, substantially improving detection accuracy in dense vegetation environments. Moreover, YOLOv8 and its subsequent versions demonstrate excellent real-time performance in detection and segmentation tasks, offering a novel technical pathway for fine-grained crop and weed recognition in agricultural fields. Currently, most crop and weed recognition studies are limited to laboratory-controlled conditions or close-range manually collected leaf images. Such datasets have limited coverage and insufficient sample diversity, making it difficult to fully represent vegetation feature distributions under complex field conditions. In contrast, UAV low-altitude remote sensing can acquire field-scale canopy imagery along preplanned flight paths, enabling efficient multi-angle and multi-temporal monitoring. However, improving the accuracy and stability of weed recognition in UAV imagery under complex field conditions remains a critical scientific challenge.

To address challenges such as morphological similarity between crops and weeds, complex field backgrounds, small target sizes, and dense distribution, this study utilizes UAV-acquired low-altitude (12 m) soybean seedling imagery as a data source and reformulates the seedling-stage weed recognition task as a semantic segmentation problem. We propose a high-precision weed segmentation method based on an improved YOLOv11-seg network. The method maintains real-time detection speed while incorporating attention enhancement modules, improved feature fusion structures, and adaptive down-sampling strategies, substantially enhancing the model’s ability to recognize and segment small weed targets in complex scenarios. The main innovations and contributions of this study include:

1. Constructed a multi-temporal UAV image dataset for the soybean seedling stage, encompassing diverse backgrounds and weed species, which provides a high-quality sample foundation for model training and generalization performance validation;
2. Proposed a task-driven model integration and improvement scheme. By incorporating the RCSOSA backbone, SEAM attention module, iRMB lightweight residual unit, and ADown adaptive downsampling module, pixel-level precise segmentation of weeds and crops was achieved. This effectively mitigates issues prevalent in traditional object detection methods, such as bounding box overlap and missed detections;
3. Designed and implemented an end-to-end engineering workflow from perception to decision-making. By integrating weed segmentation and detection results, a weed distribution map was generated based on geo-coordinate transformation and spatial interpolation. Subsequently, a variable-rate application prescription map was produced according to agronomic rules, thereby completing a full closed loop from intelligent interpretation of UAV imagery to precision operational decision-making;

This study provides visualization, data-driven analysis, and decision-support tools for precise identification and intelligent control of seedling-stage weeds at the field scale, laying the foundation for the development of an integrated “Sense–Recognize–Map–Control” precision agriculture system.

## 2 Materials and methods

### 2.1 Construction of the image dataset

#### 2.1.1 Data acquisition

All experimental data were collected from soybean experimental fields at Jianshan Farm, Heilongjiang Province. Field trials were conducted from May 30 to June 7, 2025. Image acquisition was performed between 08:00–11:00 and 13:00–18:00 under clear weather conditions with weak or light winds. Data were captured using a DJI Matrice 3M industrial UAV equipped with a high-resolution RGB camera, which was maintained perpendicular to the ground throughout the flight. The UAV flight parameters were set as follows: horizontal speed of 3.6 m/s, take-off speed of 15 m/s, flight altitude of 12 m, with a forward (along-track) overlap of 80% and side (cross-track) overlap of 70%. Images were captured at fixed time intervals in vertical mode to obtain orthophotos of the experimental area.

Using preplanned autonomous flight paths, the UAV was capable of capturing large-scale field imagery, while recording GPS coordinates for each image to provide georeferenced data for subsequent weed spatial localization. In total, 1003 images were collected, with a resolution of 5280 × 3956 pixels in JPG format.

#### 2.1.2 Dataset construction

To create a unified base map for whole-field analysis and eliminate perspective distortion from individual images, all original images with high overlap rates were mosaicked using the DJI Smart Agriculture Platform to generate a high-resolution orthophoto of the entire experimental field. This process effectively integrates redundant information from overlapping areas, resulting in a seamless two-dimensional map with consistent geometric accuracy.

To adapt to the model’s input size and construct the dataset, the orthophoto was cropped. To prevent an overestimation of the model’s generalization performance due to feature correlation between the training and test sets caused by aerial image overlap and spatial proximity, we adopted a principle of spatial geographic isolation to partition the source areas of the samples. Specifically, the field corresponding to the orthophoto was divided into several independent large regions, ensuring that the sub-images for the training, validation, and test sets were sourced from completely separate, non-overlapping areas in space. Within this framework, sub-images of 480×480 pixels were cropped using a sliding window method.

After the initial cropping, the sub-images were filtered: 246 redundant negative sample images containing no weed pixels (i.e., consisting solely of soil or shadow background) were removed to balance the positive-negative sample ratio and improve training efficiency. Ultimately, 1263 valid sub-images were retained. All sub-images were annotated at the pixel level using the LabelMe tool to segment weed regions from the background (soil, crops), generating corresponding binary mask labels.

**Figure 1:**
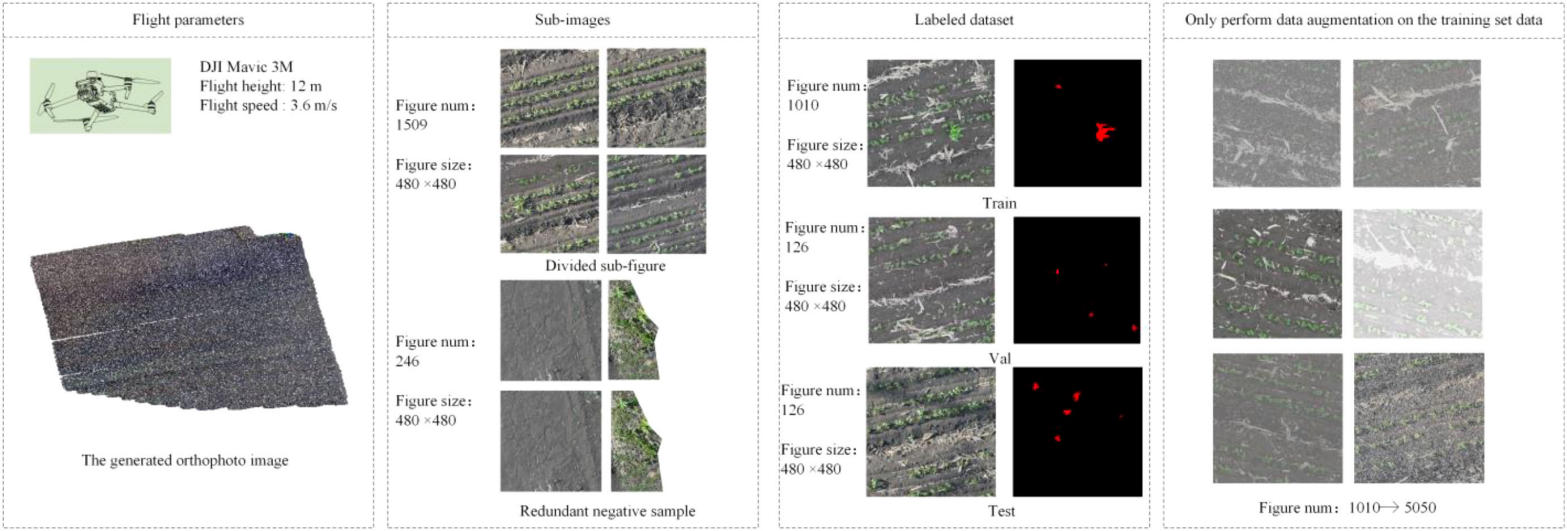
Construction of the dataset for weeds during the seedling stage.

To enhance model robustness and mitigate overfitting, data augmentation operations including random rotation, flipping, color jittering, and Gaussian noise were applied simultaneously to the training set images and their corresponding labels, expanding the number of training samples to 5050. The augmented data were then strictly partitioned according to the aforementioned geographic isolation principle into a training set (1010 original images and their augmented counterparts), a validation set (126 images), and a test set (126 images). This partitioning ensures that the model evaluation simulates the scenario of predicting entirely new, unseen areas, and the results can objectively reflect its true generalization capability. **Figure 1** illustrates the UAV data collection process and shows examples of dataset samples.

### 2.2 Bean field weed separation model

#### 2.2.1 YOLOv11-seg network

YOLOv11 continues the technical lineage of the YOLO series, supporting a variety of image processing tasks including object detection, instance segmentation, rotated object detection, and pose estimation. It offers five model scales—n, s, m, l, and x—to accommodate different scene requirements. The overall network architecture consists of three components: Input, Backbone, and Head. The Backbone is the core of the YOLO architecture, extracting multi-scale image features through stacked convolutional layers and specialized modules, and generating feature maps at different resolutions.

Key improvements in YOLOv11 include replacing the original C2f module with the C3k2 module, which optimizes computational efficiency via a Cross Stage Partial (CSP) bottleneck structure. Additionally, while retaining the Spatial Pyramid Pooling Fast (SPPF) block, a novel Cross-Stage Partial and Spatial Attention fusion module (C2PSA) is embedded afterward. In the Head, multiple C3k2 modules are employed for efficient processing and refinement of feature maps. The structure of each module is controlled by the c3k parameter: when c3k=False, a standard bottleneck structure (similar to C2f) is used; when c3k=True, it switches to the C3 module to support deeper feature extraction.

#### 2.2.2 Improved YOLOv11-seg network (RSIA-YOLOv11-seg)

**Figure 2:**
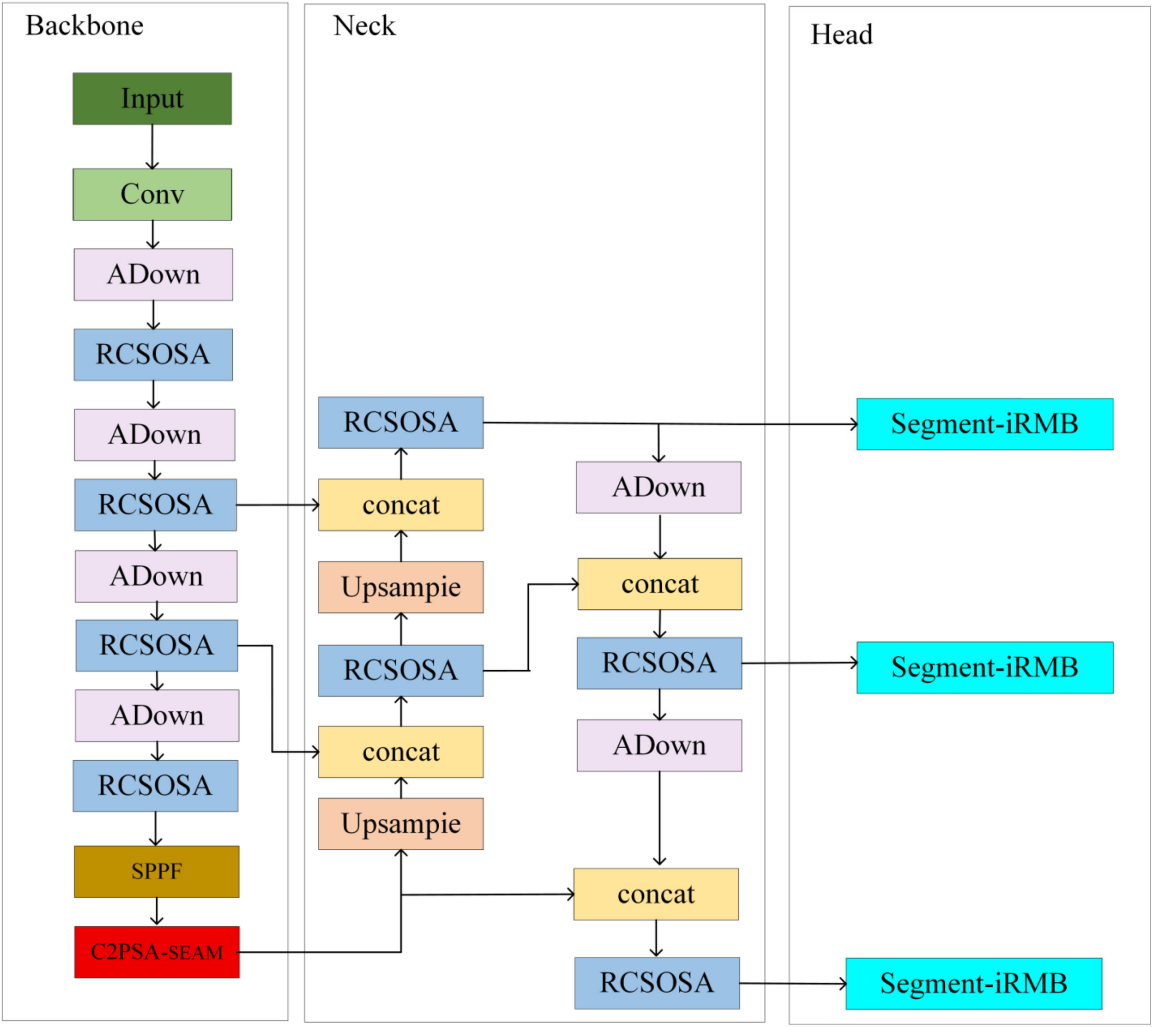
Improved YOLOv11-seg model.

To improve the accuracy and robustness of seedling-stage weed segmentation, this study systematically optimized the YOLOv11-seg architecture and introduced several module enhancements, resulting in a new model termed RSIA-YOLOv11-seg. As shown in **Figure 2**. By integrating reparameterized convolution with channel shuffle aggregation, attention enhancement modules, lightweight inverted residual structures, and adaptive down-sampling strategies into the Backbone and Neck, a modified YOLOv11-seg model was constructed with high precision, sensitivity to small targets, and computational efficiency.

The proposed model demonstrates superior feature extraction and target segmentation capabilities in complex field backgrounds and low-contrast environments, providing a reliable technical foundation for automated weed recognition and precision management under UAV remote sensing conditions. By stacking RCS modules, RCSOSA achieves feature cascading, enhancing information flow between different layers. Multi-scale feature fusion is realized through upsampling and downsampling operations, facilitating information exchange among different prediction feature layers and thereby improving detection accuracy.

#### 2.2.3 RCS-OSA module

All C3k2 modules in YOLOv11’s Backbone and Neck were replaced with RCSOSA modules, creating a feature enhancement network with richer representations and improved extraction, boosting accuracy. Standard convolutions, with fixed weights across spatial positions, reduce complexity but cannot distinguish features or adaptively weight channels and spatial regions. This limits their ability to capture long-range dependencies and spatial attention, reducing performance in complex backgrounds and multi-scale recognition.

**Figure 3:**
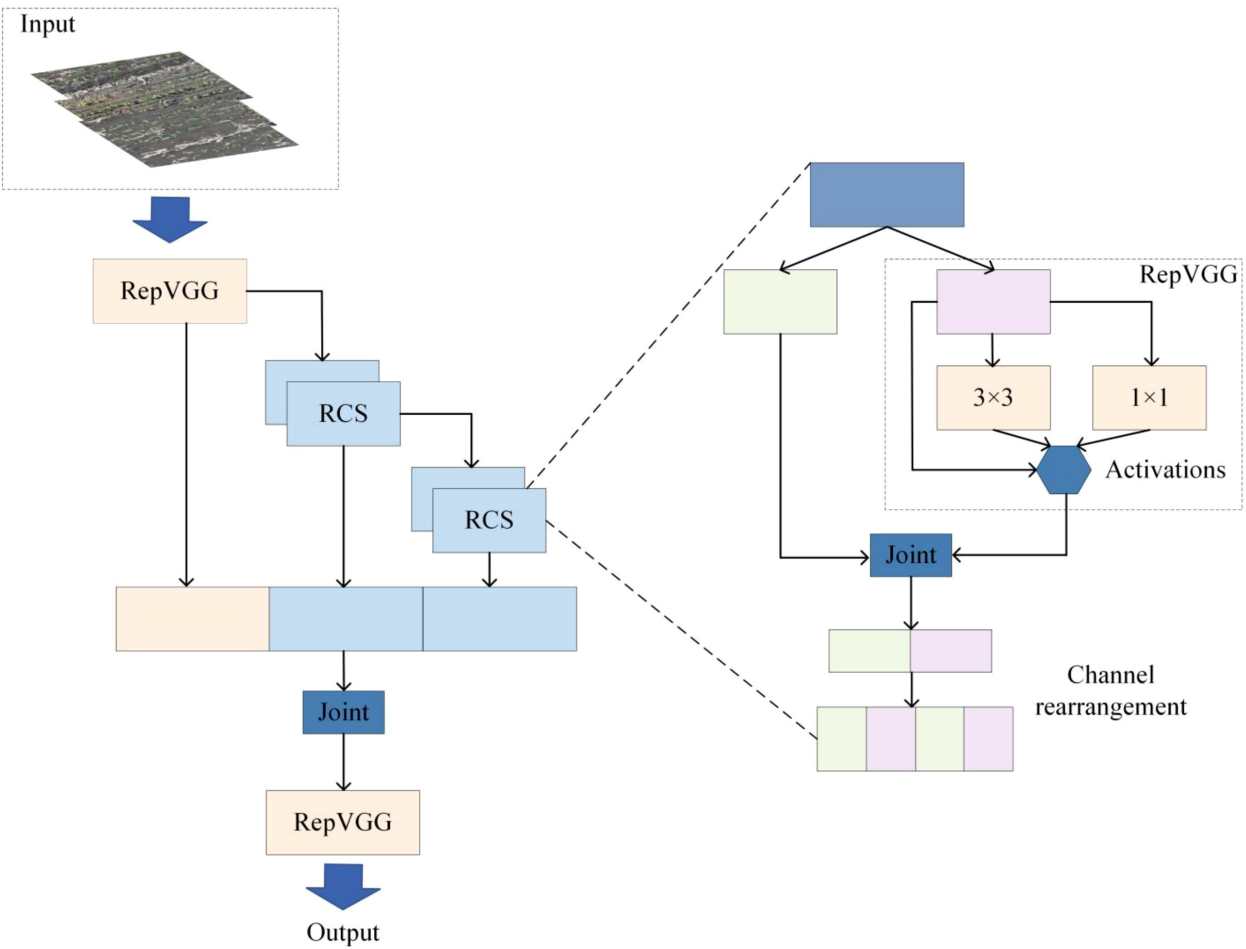
RCSOSA structure diagram.

RCSOSA (Reparameterized Convolution based on Channel Shuffle and One-Shot Aggregation) is an innovative neural network module specifically designed to enhance both the speed and accuracy of object detection tasks (Nie et al., 2024; Zhao et al., 2024). The module integrates channel shuffle and reparameterized convolution techniques (RCS) with a one-shot aggregation (OSA) strategy. During training, RCSOSA employs a multi-branch structure to learn rich feature representations, whereas during inference, structural reparameterization simplifies it into a single branch, thereby reducing memory consumption. By stacking RCS modules, RCSOSA achieves feature cascading, enhancing information flow between different layers. Multi-scale feature fusion is realized through upsampling and downsampling operations, facilitating information exchange among different prediction feature layers and thereby improving detection accuracy. The design of RCSOSA emphasizes computational efficiency, reducing memory access costs by maintaining a limited number of input and minimal output channels. Mechanistically, RCSOSA leverages a multi-branch topology to learn feature representations during training, and simplifies the architecture via reparameterization during inference to accelerate inference speed. The architecture of RCSOSA is illustrated in **Figure 3**.

Compared to the C3k2 module, RCSOSA demonstrates superior computational efficiency and enhanced feature representation capability. In terms of computational efficiency, RCSOSA significantly reduces complexity through the combination of channel shuffle and reparameterized convolution. Notably, during inference, channel splitting and shuffling operations halve the computational cost while maintaining inter-channel information exchange, enabling more efficient processing of high-dimensional features. Furthermore, by stacking RCS modules, RCSOSA not only ensures feature reuse but also enhances the flow of information across different channels between adjacent layers. This facilitates the extraction of richer features while reducing memory access overhead. Additionally, RCSOSA employs a one-shot aggregation strategy, minimizing redundant feature computation and storage requirements. This improves computational and energy efficiency while effectively integrating multi-level features, thereby enhancing the model’s capability for semantic feature extraction.

#### 2.2.4 Spatially enhanced attention module

**Figure 4:**
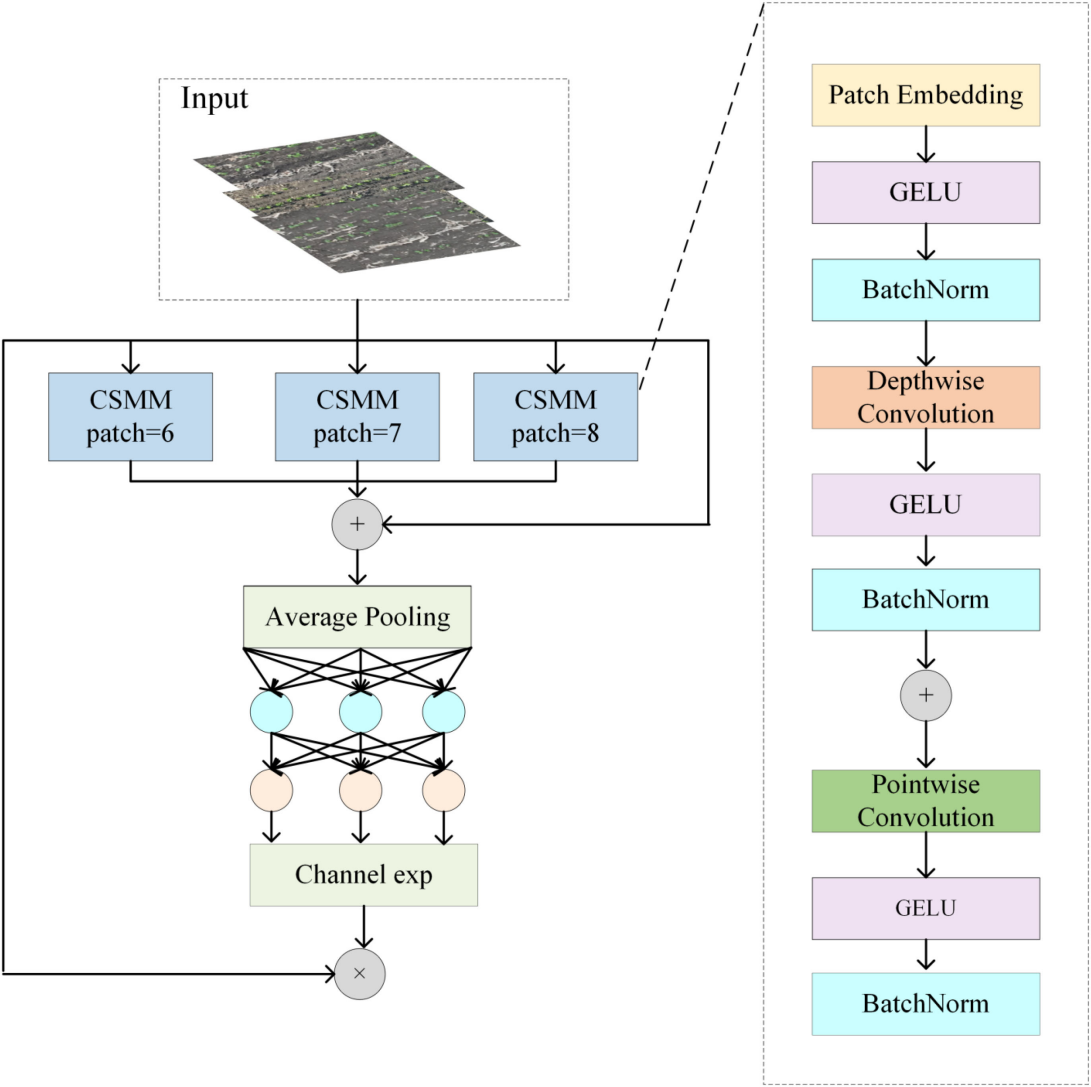
SEAM structure.

SEAM, as an innovative attention mechanism, is designed to optimize object detection performance in complex scenes (Baek et al., 2025; Caie et al., 2024; Cui et al., 2025; He et al., 2025b). By accurately distinguishing and enhancing attention, it effectively compensates for feature loss caused by occluded objects, significantly improving the model’s ability to detect such targets. The SEAM framework integrates depthwise separable convolutions, residual connections, and channel attention fully connected layers, as illustrated in **Figure 4**.

In the SEAM mechanism, channel and spatial attention work in close cooperation, assigning adaptive weights to both the channels and spatial locations of feature maps. Multi-scale features are captured by embedding patches of varying sizes, enabling the network to extract information across different spatial resolutions. Global average pooling compresses the feature maps into channel-wise vectors, which are then processed via depthwise separable convolutions performed independently on each channel. Subsequently, pointwise convolutions integrate information across channels. This approach not only reduces the number of parameters but also preserves inter-channel independence, thereby improving computational efficiency and feature extraction accuracy, allowing SEAM to more effectively capture target features in complex scenarios. Additionally, SEAM generates channel attention weights using a fully connected network, reweighting the input feature map channels to enhance the responses of important channels and improve object detection accuracy. Moreover, SEAM effectively mitigates the vanishing gradient problem and employs residual connections to facilitate training of deep networks, enhancing model stability and adaptability to targets of varying sizes (Jiang et al., 2025; Li et al., 2025b; Lu et al., 2024; Xie and Ding, 2025).

#### 2.2.5 Inverted residual mobile block with attention

In object detection and segmentation, attention mechanisms improve efficiency and accuracy, but seedling-stage weed segmentation often suffers from missed or false detections due to small, dense targets. The Inverted Residual Mobile Block with Attention (iRMB) is a lightweight mechanism designed for dense prediction, combining dynamic global modeling with static local fusion. It effectively captures features across varying weed scales, expands the receptive field, and enhances downstream performance (Xudong et al., 2024).

**Figure 5:**
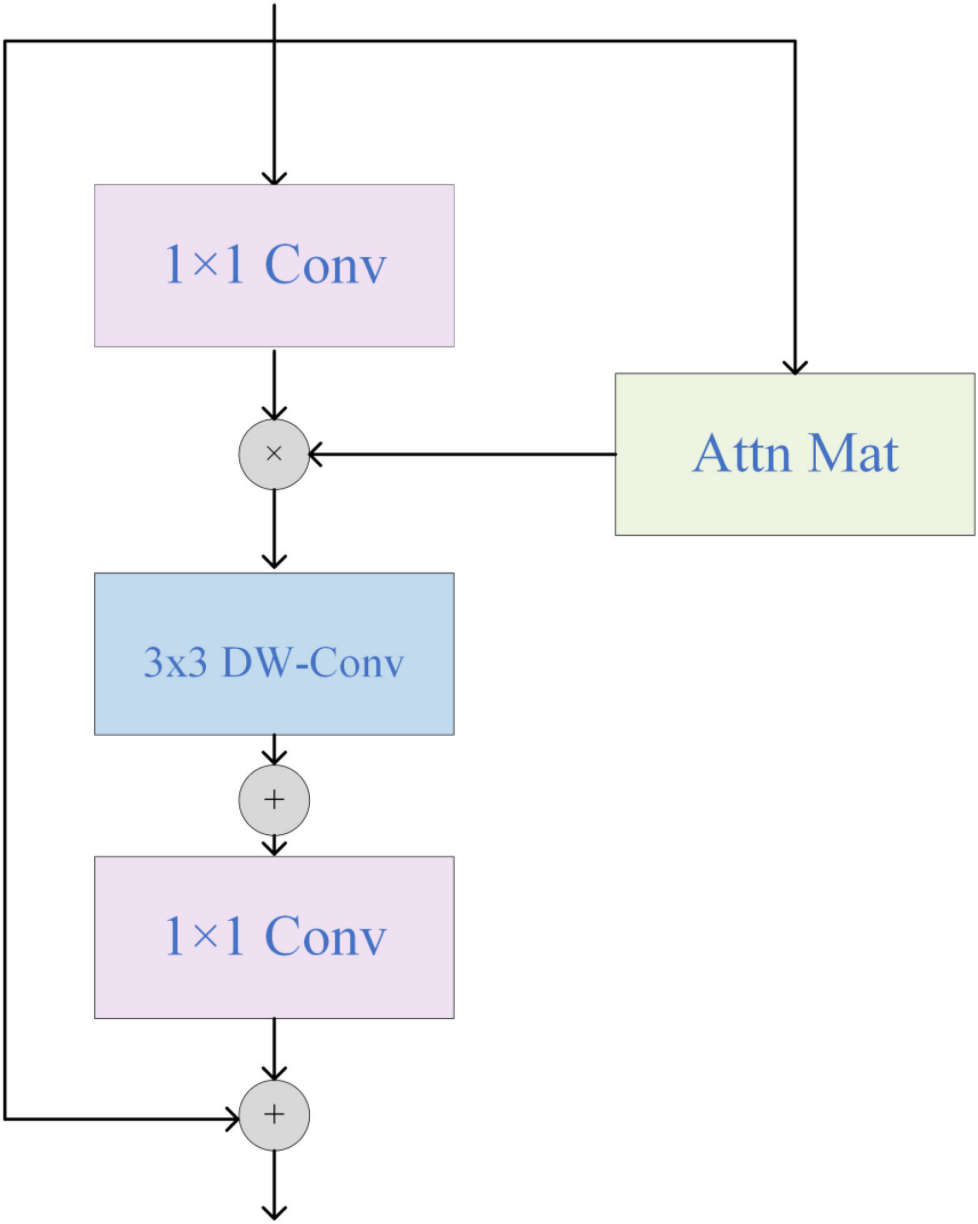
Inverted residual mobile block structure.

To enhance the YOLOv11-seg model’s ability to process large-scale information while maintaining its lightweight nature, the iRMB module was integrated into the detection head’s segmentation part. The core idea of iRMB is to leverage a lightweight CNN architecture combined with an attention-based framework to create an accurate yet computationally efficient network. iRMB integrates depthwise separable convolutions (3×3 DW-Conv) with self-attention mechanisms, unifying CNN convolution operations and Transformer-based self-attention within a single framework. By employing lightweight operators such as depthwise separable convolutions and multi-head self-attention, iRMB dynamically adjusts different expansion ratios to optimize computational resource allocation.The modular design allows flexible stacking of iRMB blocks according to task requirements, forming a ResNet-like efficient architecture. As illustrated in **Figure 5**. 1×1 convolutions are used to compress and expand channel dimensions, optimizing computational efficiency; 3×3 depthwise separable convolutions capture spatial features; and the attention mechanism captures global dependencies between features. This design enables iRMB to consider the entire input space during feature extraction, enhancing the model’s understanding of complex data and improving its robustness in dense, small-target segmentation scenarios (Huang et al., 2025; Li et al., 2025a).

#### 2.2.6 Adaptive down-sampling module

YOLOv11 typically uses strided convolutions for down-sampling, which can lose fine seedling features and reduce detection detail. To address this, the adaptive down-sampling module (ADown) from YOLOv9 was introduced and optimized in this study to replace some conventional down-sampling operations (Bai et al., 2025; Feng et al., 2025; Liao et al., 2025);.

**Figure 6:**
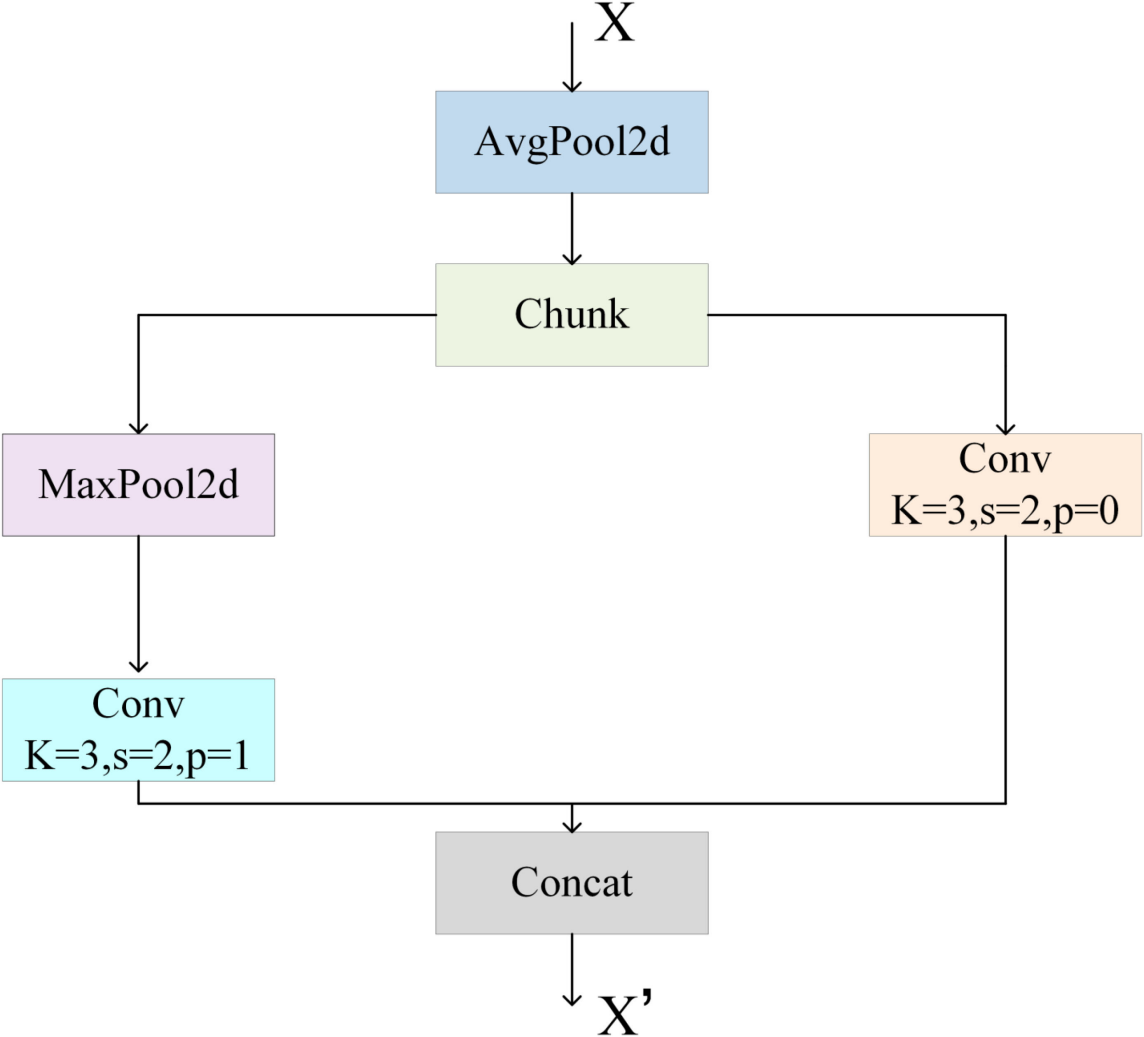
ADown network structure.

The proposed ADown module innovatively employs a dual-branch collaborative feature compression architecture, using a heterogeneous sampling strategy to efficiently reduce the feature map dimensionality while preserving discriminative information, as illustrated in **Figure 6**. Specifically, the input feature map first undergoes a 2×2 average pooling operation with a stride of 1, which effectively reduces edge effects while maintaining spatial information. This design significantly enhances the retention of small-target features, ensuring the integrity of crucial information. Subsequently, the feature map is evenly split along the channel dimension into two parts, each containing half of the channels, further reducing computational overhead.

The first path of the ADown module serves as a saliency feature enhancement pathway. The sub-features are first processed by a 3×3 max-pooling operation (stride = 2, padding = 1), which emphasizes salient regions by capturing local maxima while reducing redundant information. This is followed by a 1×1 convolution (stride = 1) for channel reorganization, enhancing cross-channel feature correlations and fine-detail extraction. This design not only reduces computational complexity but also effectively preserves subtle image features.

The second path functions as a fine-grained feature learning pathway. Here, sub-features are spatially down-sampled using a 3×3 convolution (stride = 2), achieving dimensionality reduction of the feature map. Due to the small kernel size and stride, this pathway efficiently reduces spatial dimensions while extracting detailed local features.

By splitting the input feature map and processing it through these two pathways, the ADown module successfully optimizes the computational load along each path. Compared to conventional single-convolution operations, the combination of channel splitting and pooling effectively reduces the number of convolution parameters, thereby decreasing overall computational overhead. Specifically, each branch’s input and output channels are halved, resulting in a significant reduction of convolutional layer parameters. Assuming the input feature map has dimensions h×w×c and the down-sampled feature map has dimensions h/2×w/2×c, the parameter count P_C_ and computational complexity F_C_ of the 3×3 convolution with stride 2, as well as the corresponding metrics for the ADown module, can be mathematically expressed as follows:

(1) $$\{\begin{array}{l} P_{a}=\frac{5}{2}c^{2}\\ F_{a}=\frac{5}{3}c^{2}\times h\times w \end{array}$$

(2) $$\{\begin{array}{l} P_{a}=9c^{2}\\ F_{a}=\frac{9}{4}c^{2}\times h\times w \end{array}$$

Based on the analyses presented in Equations 1 and 2, the parameter count and computational complexity of traditional strided convolution with a stride of 2 are approximately 3.6 times higher than those of the ADown module. The stability and efficiency of the ADown module stem from its dual-path complementary design. The depthwise separable convolution path preserves rich spatial detail features, while the max-pooling path captures strong semantic cues. The orthogonal fusion of these two pathways effectively mitigates feature degradation. Furthermore, by leveraging the concept of structural re-parameterization, the ADown module reduces model complexity while maintaining robust detection of multi-scale targets.

## 3 Experimental results and analysis

### 3.1 Experimental environment and training parameter settings

To ensure the fairness of ablation and comparative experiments, all models—including YOLOv8s-seg, YOLOv11s-seg, YOLOv11n-seg, YOLOv12s-seg, and the proposed model—were trained and evaluated under identical conditions. Specifically, the experiments were conducted on a hardware platform consisting of an Intel(R) Core(TM) i5-13490F CPU and an NVIDIA RTX 5060 Ti GPU, running the Windows 10 operating system. The software environment was based on Python 3.8.10 and the PyTorch framework. A unified training protocol was adopted: the input image resolution was fixed at 480×480; the SGD optimizer was used with an initial learning rate of 0.01; the batch size was set to 16 for a total of 300 training epochs. All models were initialized with their official pre-trained weights and employed the same data augmentation strategy, along with an 8:1:1 split of the dataset into training, validation, and test sets. This configuration ensures that performance differences are primarily attributable to improvements in the model architecture.

### 3.2 Model evaluation

To select the optimal model, performance metrics included detection precision (Precision(Box)), segmentation precision (Precision(Mask)), detection recall (Recall(Box)), segmentation recall (Recall(Mask)), mean average precision for detection (mAP@0.5(Box)), and mean average precision for segmentation (mAP@0.5(Mask)). These metrics comprehensively evaluate the model’s detection and segmentation performance, as defined in Equations 3–5. The mean average precision (mAP) represents the average AP across all categories and serves as an overall measure of the object detection algorithm’s performance. Precision quantifies the ratio of correctly classified positive samples to all predicted positives, reflecting the model’s accuracy. Recall measures the number of correctly identified positives among all actual positives, indicating the model’s effectiveness in capturing all relevant targets within the dataset.

(3) $$mAP=\frac{\sum_{i=1}^{N}AP_{i}}{N}AP$$

As shown in Equation 3, the mean Average Precision (mAP) is an indicator that measures the average precision across all categories. Here, N denotes the total number of object detection categories, APi represents the Average Precision for the i-th category, and mAP@0.5 specifically refers to the mAP value when the Intersection over Union (IoU) threshold is set to 0.5.

(4) $$\text{Precision }=\frac{TP}{TP+FP}$$

As shown in Equation 4, precision reflects the accuracy of positive predictions made by the model, representing the percentage of true positives among all samples detected as positive. Specifically, TP in the equation denotes the number of correctly identified positive samples, while FP corresponds to the number of negative samples that were falsely reported as positive.

(5) $$\text{Recall }=\frac{TP}{TP+FN}$$

The formula for Recall is shown in Equation 5, defined as the proportion of actual positive samples that are correctly predicted by the model. Here, TP is the number of correctly identified positive samples, and FN denotes the number of positive samples that were incorrectly classified as negative.

### 3.3 Results analysis

#### 3.3.1 Ablation study

To evaluate the impact of each module on model performance, ablation studies were conducted, with the results presented in **Table 1**. Test 1 represents the baseline model, achieving a detection mAP@0.5(Box) of 0.77 and a segmentation mAP@0.5(Mask) of 0.719. The introduction of the RCSOSA module (Test 2) yielded the most substantial performance gains. The detection recall (R-Box) surged from the baseline of 0.687 to 0.805 (a relative improvement of 17.2%), while the precision (P-Box) increased only marginally from 0.830 to 0.865. This clearly demonstrates that RCSOSA, through its cross-scale context aggregation capability, significantly expands the model’s feature perception field. Its primary effect is a marked reduction in missed detections of small, marginal, or background-blended weeds. The substantial improvements in mAP (Box: 0.77→0.884; Mask: 0.719→0.813) are also largely attributable to the gain in recall.

**Table 1:** Comparison of ablation experiment accuracy.

Test umber	RCS0SA	SEAM	iRMB	Adown	P(%)-Box	R(%)-Box	mAP@0.5(%)-Box	P(%)-Mask	R(%)-Mask	mAP@0.5(%)-Mask	GFLOPs (GB)	Parameters (M)
1					0.830	0.687	0.77	0.781	0.639	0.719	10.2	2.83
2	**√**				0.865	0.805	0.884	0.83	0.752	0.813	25.4	8.4
3		**√**			0.813	0.718	0.812	0.788	0.658	0.738	9.5	2.80
4			**√**		0.797	0.709	0.81	0.788	0.642	0.736	10.3	3.18
5				**√**	0.831	0.774	0.852	0.793	0.699	0.769	8.4	2.35
6	**√**	**√**			0.876	0.809	0.89	0.854	0.754	0.830	25.4	8.4
7	**√**	**√**	**√**		0.883	0.798	0.884	0.86	0.748	0.825	26.1	8.76
8	**√**		**√**	**√**	0.902	0.804	0.895	0.856	0.755	0.823	25.3	8.37
9	**√**	**√**		**√**	0.880	0.810	0.891	0.893	0.756	0.819	24.6	8.0
10	**√**	**√**	**√**	**√**	0.883	0.807	0.89	0.868	0.760	0.840	25.3	8.3

Incorporating the SEAM module alone (Test 3) provided limited improvements to the detection metrics. However, its combination with RCSOSA (Test 6) proved highly instructive: building upon the high-recall features provided by RCSOSA, the addition of SEAM further increased segmentation precision (P-Mask) from 0.830 to 0.854. This confirms the core function of SEAM: by leveraging channel and spatial attention mechanisms, it enhances the model’s ability to discriminate between foreground (weeds) and background (soil, crops), thereby generating more accurate boundaries in dense and overlapping regions and effectively suppressing false segmentations. The iRMB module (Test 4) contributed to a balanced, slight increase in both precision and recall, indicating that its improved feature fusion mechanism strengthens the discriminative power of the detection head. The ADown module (Test 5) demonstrated exceptional efficiency advantages: while reducing the computational cost (GFLOPs) from 10.2G to 8.4G (a 17.6% reduction), it actually increased mAP@0.5(Box) from 0.770 to 0.852. This verifies that its adaptive downsampling strategy effectively preserves crucial details, avoiding the information loss common with conventional downsampling in low-contrast scenarios.

The module combination experiments clearly demonstrate synergistic effects. The “RCSOSA + SEAM” pair (Test 6) forms the performance core, integrating “high-recall coverage” with “high-precision segmentation, “ and achieved excellent overall metrics (mAP@0.5(Box)=0.890). Building upon this foundation, introducing ADown (Test 9) allowed for a significant reduction in computational load (GFLOPs decreased from 25.4G to 24.6G) while nearly maintaining equivalent performance. Ultimately, the full model (Test 10), which integrates the advantages of all modules, achieved the best balance among recall (R-Box: 0.807), segmentation precision (P-Mask: 0.868), and efficiency (25.3 GFLOPs, 8.3 M Params), attaining the highest performance with an mAP@0.5(Mask) of 0.840.

**Figure 7:**
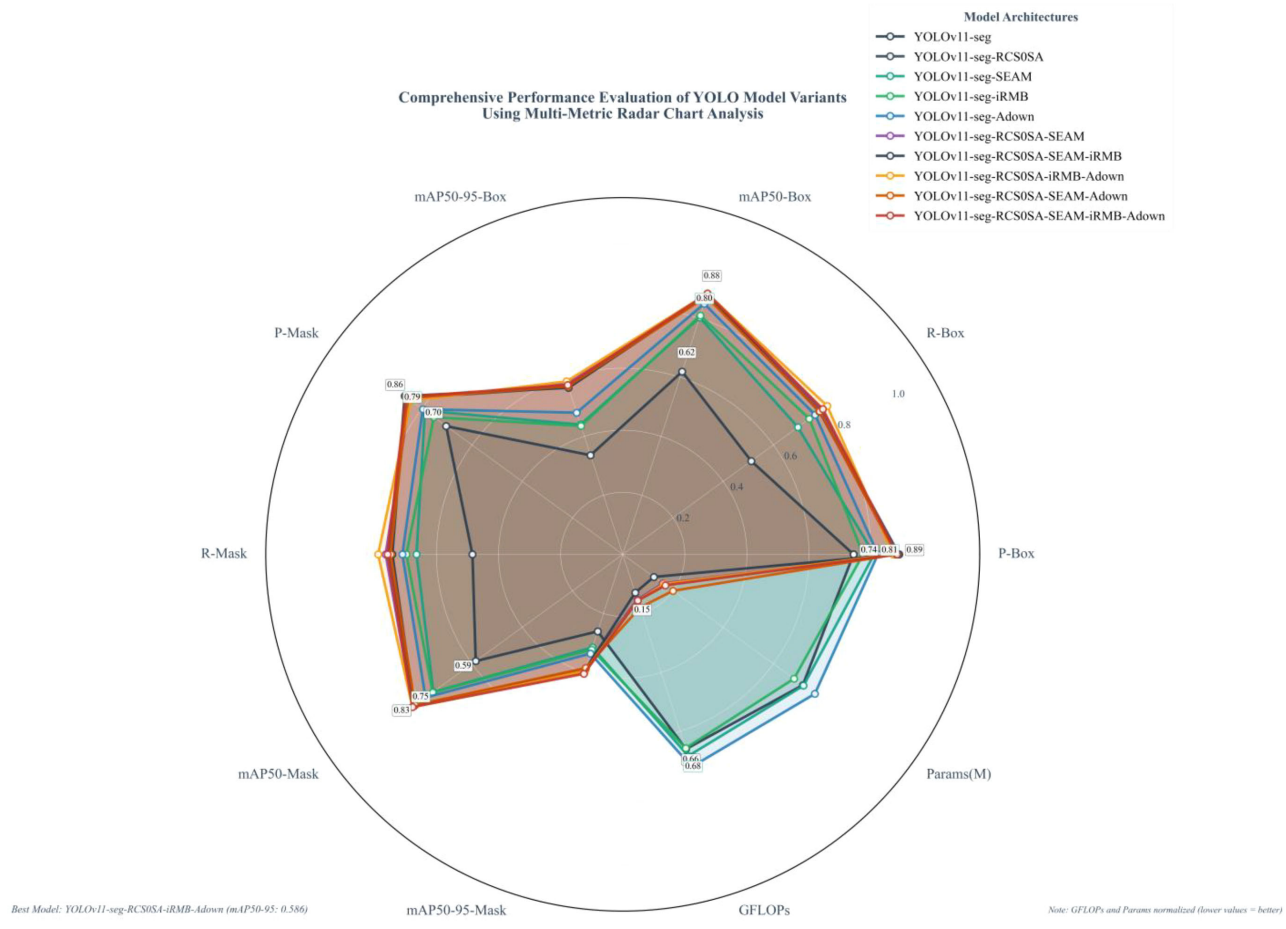
Ablation study radar chart.

To more intuitively illustrate the comprehensive impact of different modules on model performance, a multi-index normalized radar chart was plotted (as shown in **Figure 7**). The radar chart visualizes the results of each experimental group across nine dimensions, including detection precision, recall, mAP@0.5, mAP@0.5–0.95, segmentation precision, computational complexity (GFLOPs), and parameter count (Params). In the chart, moving from the center outward represents a trend from weaker to stronger performance, and the overall contour of the radar chart reflects the model’s comprehensive performance across these multiple dimensions.

As can be seen from **Figure 7**, the radar contour of the baseline YOLOv11-seg model is relatively contracted. With the introduction of the RCSOSA, SEAM, iRMB, and ADown modules, the contour progressively expands outward, and the overall shape becomes fuller. Among them, the RCSOSA module shows the most significant expansion along the detection precision axis. The SEAM and iRMB modules strengthen the segmentation-related dimensions, while the ADown module maintains high accuracy while controlling computational cost, resulting in a more balanced distribution of the contour. Ultimately, the model integrating all four modules forms the outermost closed shape, indicating that it achieves the optimal balance among detection and segmentation accuracy, efficiency, and model complexity. The radar chart results visually confirm the significant contribution of the multi-module design to overall performance enhancement.

**Figure 8:**
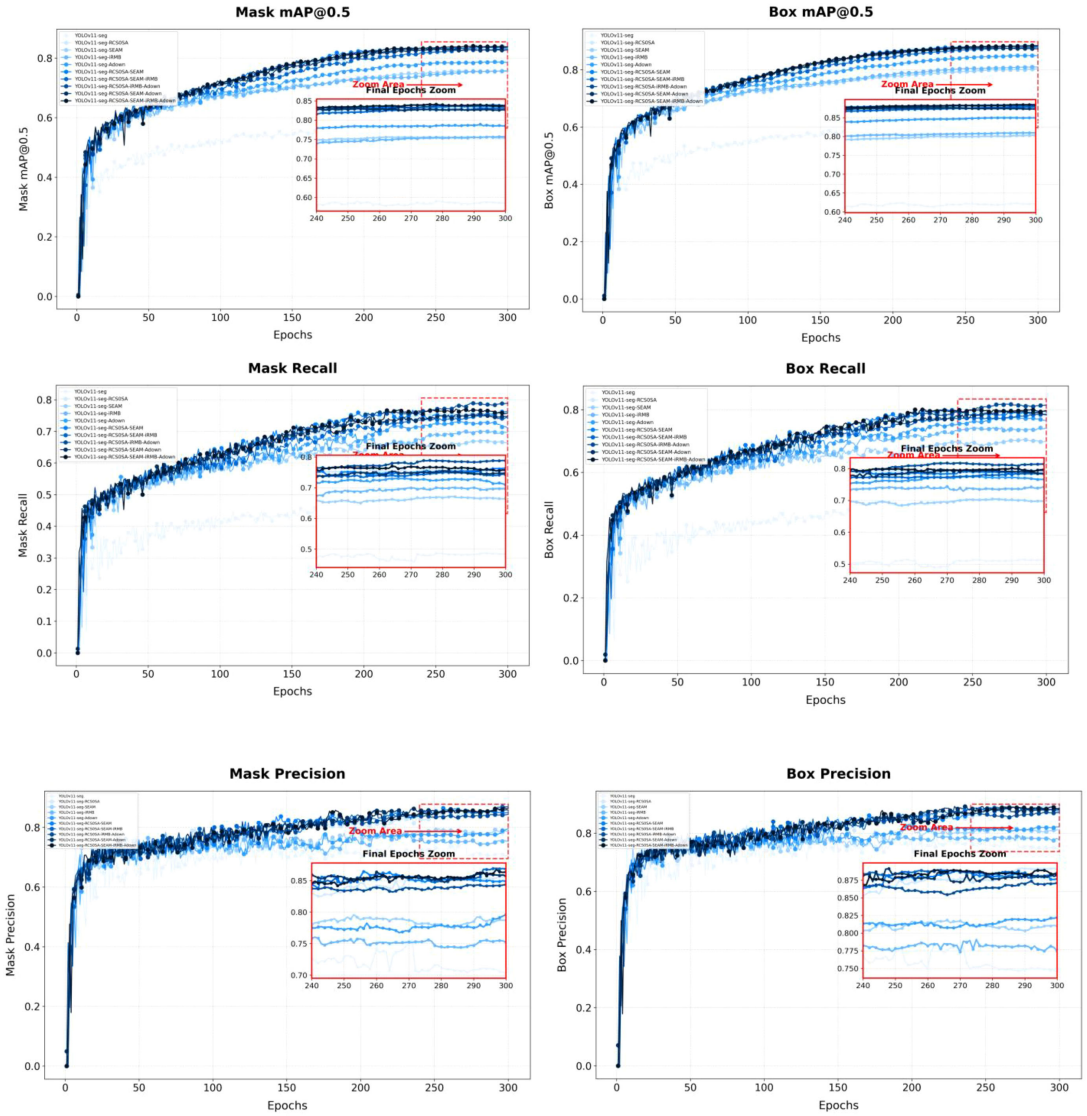
Ablation study results curves.

As shown in **Figure 8**. the curves of the ablation experiments demonstrate the effectiveness and synergistic contributions of the proposed modules. By integrating low-altitude UAV remote sensing, intelligent recognition, and GIS-based spatial analysis, this study achieves a fully automated and intelligent workflow from image acquisition and target detection to spatial mapping and variable-rate herbicide application, providing a feasible technical pathway for precision agriculture.

**Figure 9:**
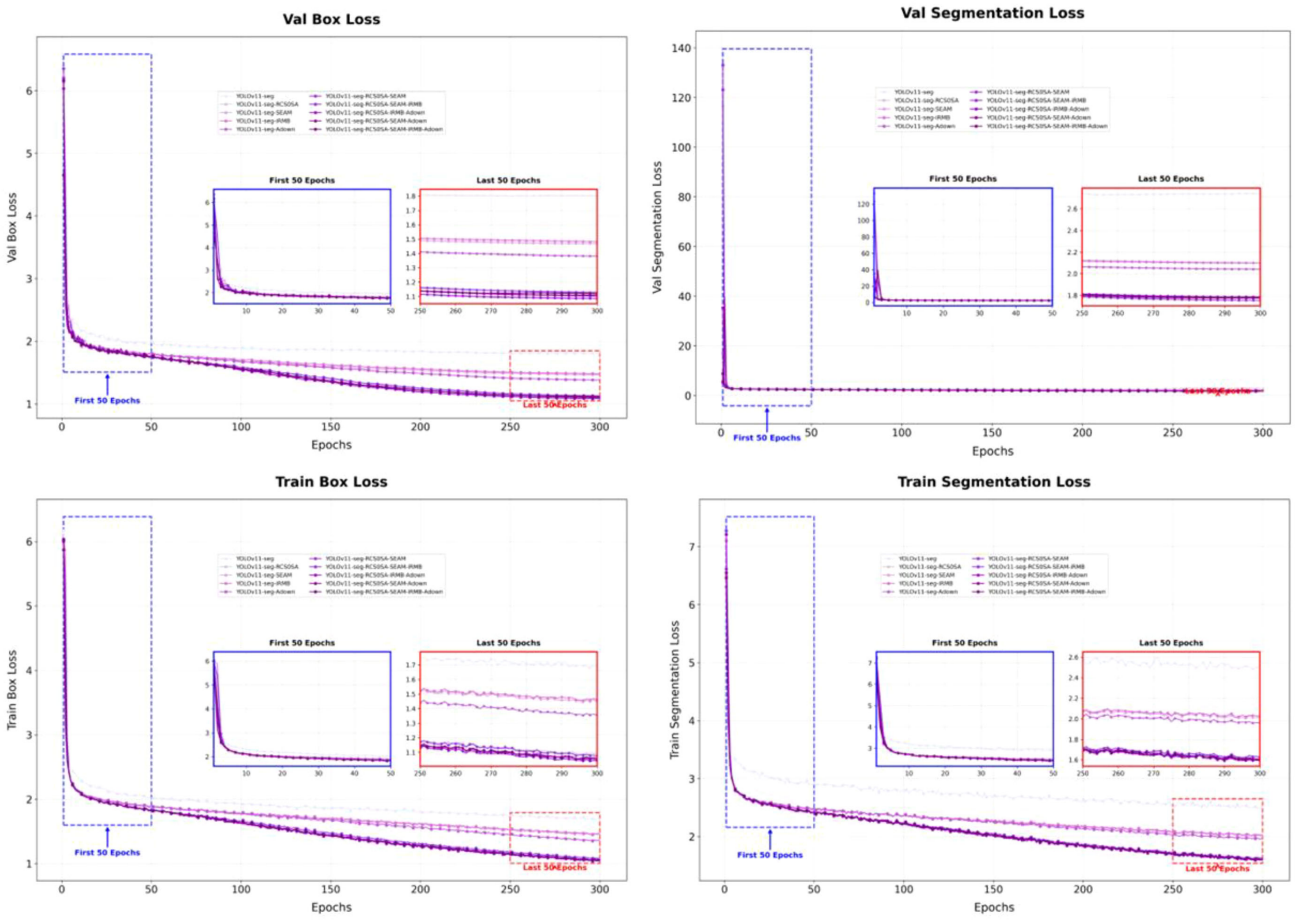
Training and validation loss curves of the models.

As shown in **Figure 9** the training and validation loss curves of the models in the ablation experiments are presented to visually compare the effects of different structural improvements on model convergence. It can be observed that all models exhibit a rapid decrease in loss during the initial training phase, followed by a stable convergence stage, with a relatively small gap between training and validation losses, indicating no significant overfitting or underfitting. As the number of training epochs increases, the loss curves gradually stabilize and converge to similar levels, demonstrating that all models possess strong learning and generalization capabilities on the constructed soybean field weed dataset.

Notably, the proposed improved model demonstrates superior convergence speed and stability compared to the baseline. Benefiting from the efficient feature aggregation of the RCSOSA module, the spatial attention enhancement of the SEAM module, the lightweight feature representation of the iRMB structure, and the adaptive sampling strategy of the ADown module, the model achieves faster gradient descent and smoother convergence in the initial stages. The validation loss decreases more rapidly with reduced fluctuation, indicating that the improved model can more effectively capture critical feature information in complex backgrounds and dense small-object scenarios, resulting in better fitting performance.

Overall, the enhanced YOLOv11-seg model exhibits stable convergence and strong generalization during training, validating the rationality and robustness of the network design. This reliable training performance provides a solid algorithmic and data foundation for subsequent high-precision segmentation of soybean seedling-stage weeds, spatial distribution mapping, and variable-rate herbicide application decision-making.

#### 3.3.2 Comparative experiments with different models

The experimental results are summarized in **Table 2**, showing that the proposed improved model performs excellently in both weed detection and segmentation tasks in soybean fields. For the object detection task, the improved model achieved P(%)-Box =0.883, R(%)-Box =0.807, and mAP@0.5(Box) = 0.890, representing improvements of approximately 4.6% and 8.5% in mAP compared to YOLOv11s-seg and YOLOv12s-seg, respectively. In the instance segmentation task, the model attained P(%)-Mask = 0.868, R(%)-Mask = 0.760, and mAP@0.5(Mask) = 0.840, also outperforming the other comparison models. These results indicate that the improved model achieves higher accuracy in both localization and segmentation of weed targets.

**Table 2:** Algorithm comparison of test results.

Model	P(%)-Box	R(%)-Box	mAP@0.5(%)-Box	P(%)-Mask	R(%)-Mask	mAP@0.5(%)-Mask	GFLOPs (GB)	Parameters (M)
YOLOv8s-seg	0.913	0.80	0.876	0.859	0.753	0.824	39.9	11.78
YOLOv11n-seg	0.830	0.687	0.770	0.781	0.639	0.719	10.2	2.83
YOLOv11s-seg	0.872	0.796	0.844	0.828	0.730	0.806	32.8	10.07
YOLOv12s-seg	0.854	0.746	0.820	0.859	0.678	0.781	32.7	9.89
Ours	0.883	0.807	0.89	0.868	0.760	0.840	25.3	8.34

**Figure 10:**
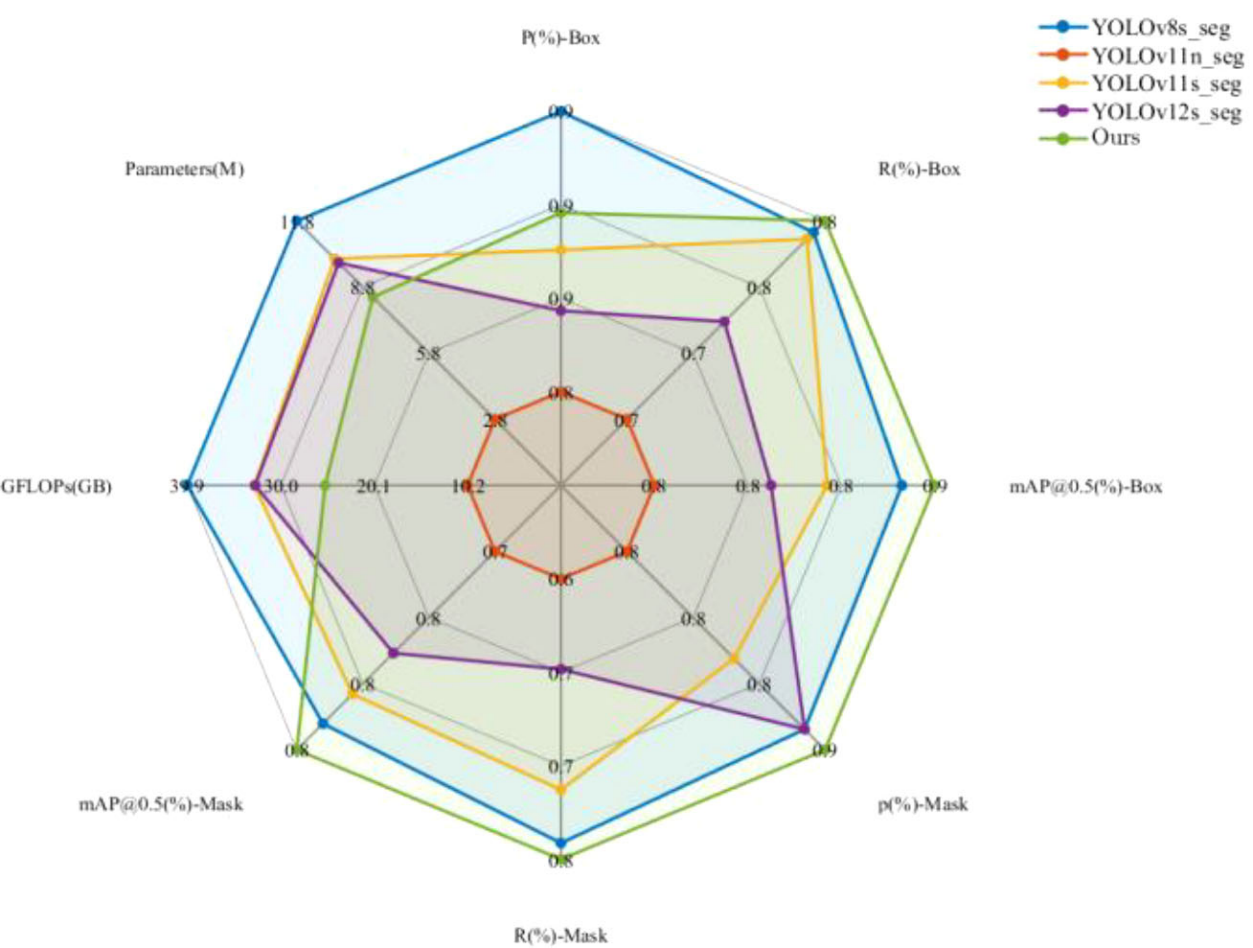
Performance comparison of different YOLO models on the radar chart (normalized data: the higher the value, the better the performance from the center to the periphery).

To further illustrate the comprehensive performance differences, a multi-metric normalized radar chart was generated (as shown in **Figure 10**). The radar chart integrates key performance indicators—including detection precision, recall, mAP, segmentation metrics, parameter count (Params), and computational complexity (GFLOPs)—to visualize the overall capability of each model. Each axis represents a standardized metric, with values farther from the center indicating stronger performance.

As depicted in **Figure 10** the proposed model forms the outermost and most balanced contour, reflecting its well-rounded performance across detection, segmentation, and efficiency dimensions. In contrast, YOLOv8s-seg and YOLOv11s-seg exhibit relatively good detection accuracy but weaker segmentation or computational efficiency, while YOLOv12s-seg shows partial improvements yet remains uneven overall. The proposed model achieves the best trade-off between accuracy and complexity, with the highest values in both mAP@0.5(Box) and mAP@0.5(Mask). This visual evidence strongly supports the effectiveness of the proposed architectural enhancements and module synergy in improving multi-scale feature extraction, spatial perception, and fine-grained segmentation accuracy.

**Figure 11:**
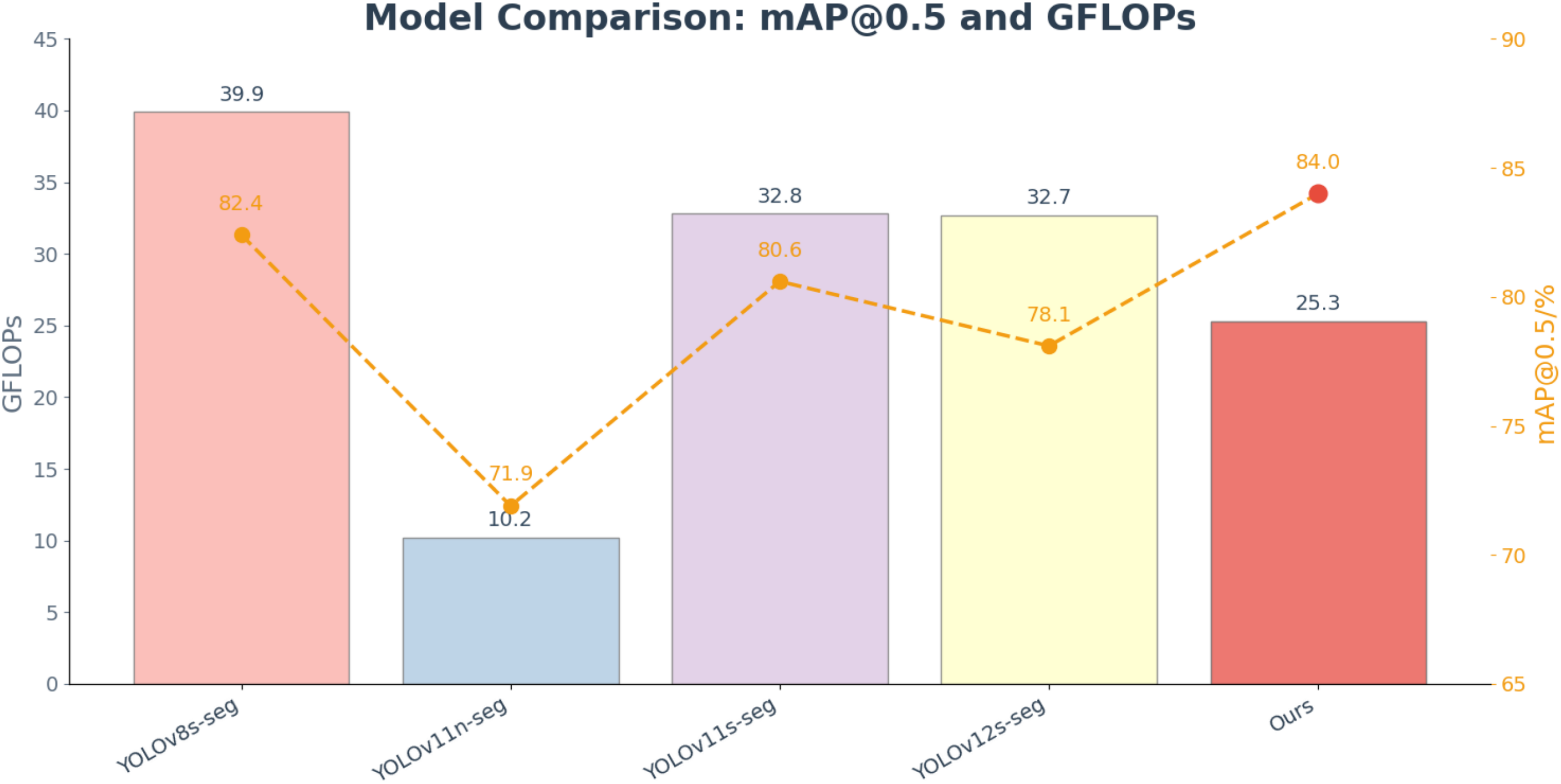
Comparison of mAP@0.5 and GFLOPs among mainstream YOLO segmentation models.

As shown in **Figure 11**. Although the model’s GFLOPs and parameter count have increased slightly (25.3 GB and 8.34 M), it still maintains a clear lightweight advantage compared to YOLOv8s-seg (39.9 GB, 11.78 M). Therefore, this study moderately increased the model complexity while ensuring high accuracy to meet the experimental precision requirements. The final model successfully achieved the experimental objectives for weed detection and segmentation in soybean fields. Based on the high-precision weed information extracted by this model, high-resolution spatial distribution maps of weeds were further generated, and variable-rate herbicide application decision maps were constructed accordingly, providing a scientific basis for large-scale precision spraying and realizing an integrated “sense–recognize–control” workflow for field weed management.

#### 3.2.3 Segmentation and detection performance of the RSIA-YOLOv11-seg model

**Figure 12:**
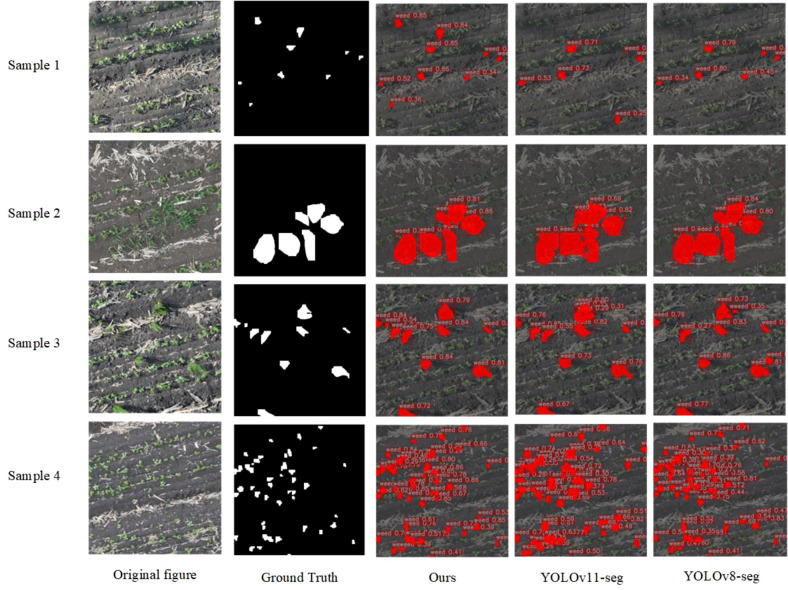
Comparative visualization of segmentation results produced by different models.

**Figure 13:**
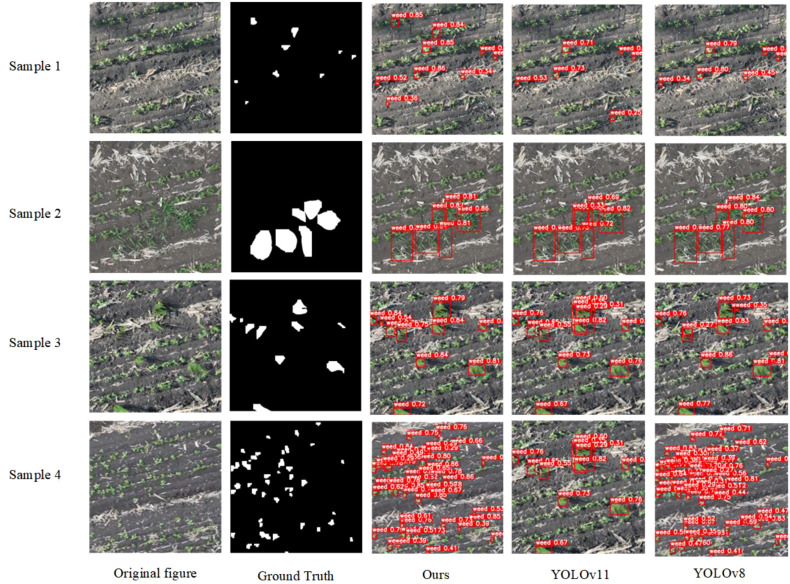
Comparative visualization of detection results produced by different models.

To further validate the effectiveness of the proposed model, detection and segmentation results were visualized using low-altitude (12 m) UAV-acquired images of soybean fields during the seedling stage, as shown in **Figures 12** and **13**. Comparative analysis with YOLOv11(-seg) and YOLOv8(-seg) models allows for an intuitive evaluation of the improved model’s recognition and segmentation performance in complex field environments.

As illustrated in **Figure 12** the segmentation masks generated by RSIA-YOLOv11-seg closely match the ground truth annotations, accurately delineating the morphological boundaries of weed targets. The improved model demonstrates stronger discrimination and localization capabilities, particularly for broadleaf weeds with morphological similarity to soybean seedlings, densely packed small weeds, or partially overlapping targets. Compared to YOLOv8-seg and YOLOv11-seg, RSIA-YOLOv11-seg produces smoother boundaries and more complete regions, significantly reducing under-segmentation and mis-segmentation, thereby effectively enhancing spatial precision in target segmentation.

The detection results in **Figure 13** further confirm the superior performance of the improved model. It outperforms the comparison models in both bounding box localization and confidence scores, accurately identifying weeds of various scales while maintaining stable detection under challenging conditions such as complex backgrounds, shadow interference, and uneven illumination. This demonstrates the model’s robustness and generalization ability.

The higher-precision segmentation results not only improve weed recognition accuracy but also provide more reliable boundary references for subsequent coordinate transformation and spatial mapping. The precise segmentation allows for more accurate georeferencing of weed targets, thereby enhancing the accuracy of weed spatial distribution mapping and variable-rate herbicide application zones.

In summary, the improved model exhibits significant advantages in both detection and segmentation tasks, enhancing recognition accuracy, boundary consistency, and adaptability in complex field environments. By integrating UAV remote sensing imagery with GIS-based spatial analysis, the model enables a fully automated and intelligent workflow from image acquisition and target recognition to weed spatial localization and variable-rate herbicide decision-making, providing a reliable technical foundation for precision agriculture.

Note: In the figure, the red area represents weed pixels, and the gray area represents background pixels.

#### 3.2.4 Coordinate transformation and weed spatial mapping

To accurately map weed targets from image to geographic space, a multi-coordinate transformation framework was established, spanning pixel, image physical, camera, world, and WGS-84 geodetic systems. Weed pixel coordinates were first converted to physical coordinates using camera intrinsic parameters, then mapped into 3D camera coordinates based on the pinhole model and UAV flight height.By integrating high-frequency UAV attitude (pitch, roll, yaw) and POS positional data, a rotation-translation matrix transformed camera coordinates into ground-referenced world coordinates. Finally, centimeter-level RTK data enabled precise conversion to the global geodetic system via the Gauss-Krüger projection.

The innovation of this coordinate transformation chain lies in the seamless integration of photogrammetry, computer vision, and precision agriculture requirements. By tightly coupling multi-source sensor data, it effectively addresses the challenge of unifying visual perception and geographic spatial information in UAV remote sensing, providing a critical spatial reference for generating high-precision weed distribution maps and implementing variable-rate applications (Hu et al., 2024).

This study developed a high-resolution weed spatial distribution mapping method for precision pesticide application. The specific procedure is as follows:

1. Image Acquisition and Preprocessing

High-resolution RGB images were acquired using an unmanned aerial vehicle (UAV) over a 253.55-acre experimental soybean field. The images were then mosaicked, fused, and orthorectified using the DJI Agricultural Intelligence Platform to generate a high-resolution orthophoto with precise georeferencing, providing foundational data for subsequent spatial analysis.

2. Analytical Grid and Operational Unit Alignment

Considering the 12-meter effective working width of the ground sprayer, the entire field was uniformly partitioned into standard rectangular grids measuring 12 m × 12 m. This grid size perfectly aligns with both the analytical and application units, ensuring that the spatial quantification results can be directly used to guide variable-rate operations, thereby guaranteeing uniform and precise pesticide application.

3. Intelligent Weed Identification and Georeferencing

The improved YOLOv11n-seg model was employed to identify and segment weeds within the orthophoto. This model exhibits enhanced capability in detecting small weeds and delineating their boundaries. Leveraging the GPS metadata embedded in the UAV data, precise longitude and latitude coordinates were obtained for each identified weed, achieving the localization of weeds from image space to geographic space.

4. Spatial Aggregation and Density Classification

Within the ArcGIS Pro platform, the georeferenced weed points were spatially joined to their corresponding 12 m × 12 m grids. The number of weeds within each grid was counted to complete the spatial aggregation. Subsequently, the weed density per grid was scientifically classified into five levels (1–24, 25–48, 49–72, 73–96, and ≥97 weeds/grid) using the Natural Breaks (Jenks) method, informed by local plant protection expertise. The classification criteria are detailed in **Table 3**.

**Table 3:** Weed density classification criteria.

Density tier	Weed count range
1	1-24
2	25-48
3	49-72
4	73-96
5	≥ 97

5. Thematic Map Generation and Prescription Decision-Making

**Figure 14:**
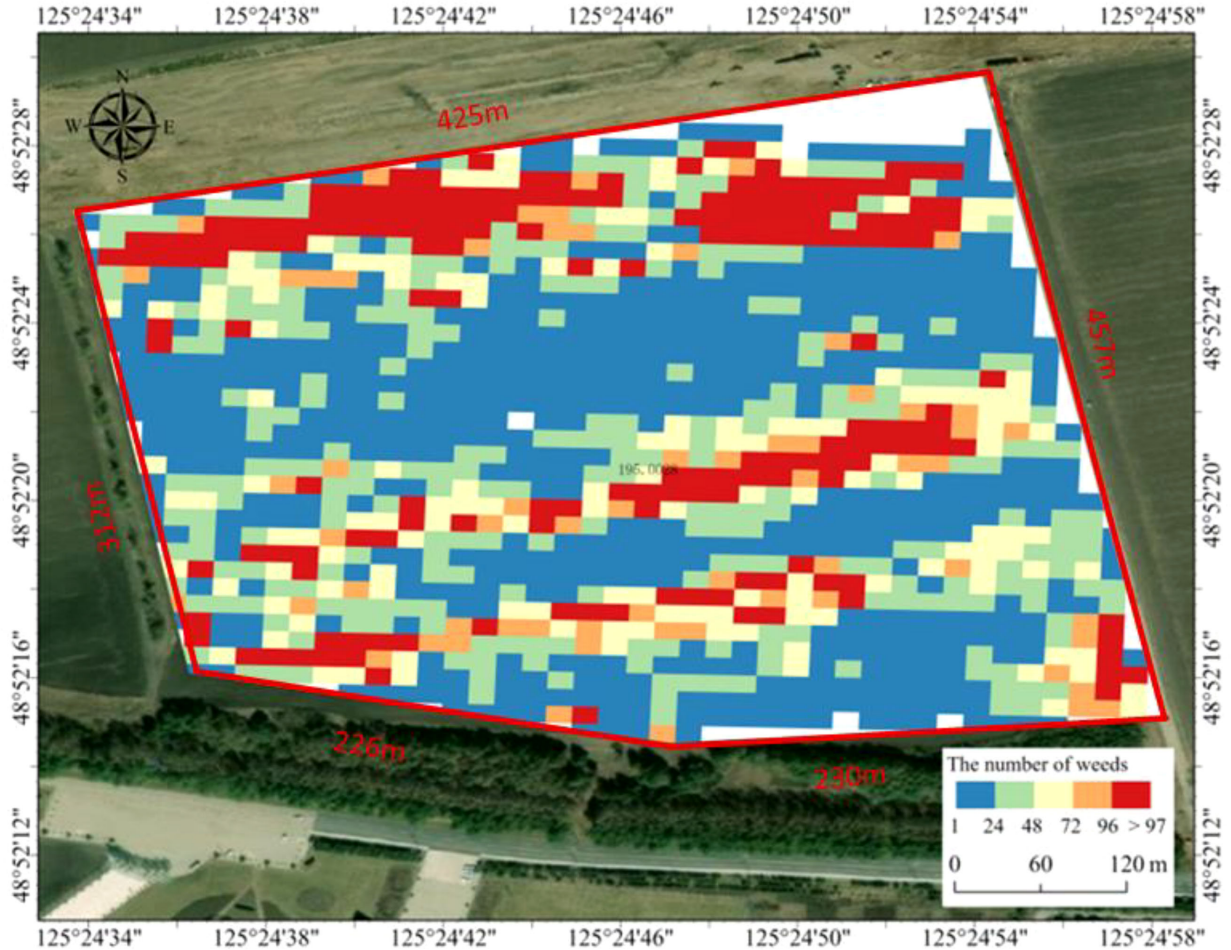
Large-scale field weed distribution map.

Based on the density classification results, an intuitive weed spatial distribution thematic map was generated using color coding (**Figure 14**), which includes standard map elements such as a north arrow and scale bar. This thematic map essentially serves as a prescription map precisely aligned with the sprayer’s working width. It enables intelligent decision-making regarding herbicide type and application rate for each grid based on its weed density level. This approach reduces pesticide use in low-density areas while ensuring control efficacy in high-density areas, facilitating precise, environmentally conscious variable-rate spray operations.

#### 3.2.5 Generation of variable-rate herbicide maps

Chemical control of soybean stem-and-leaf weeds is typically divided into the true leaf and compound leaf stages. According to the Critical Period of Weed Competition (CPWC) theory, soybeans at the true leaf stage grow vigorously and can outcompete small weeds. As weeds grow, competition intensifies, so early-stage variable-rate herbicide application can suppress weeds while ensuring crop safety.

**Figure 15:**
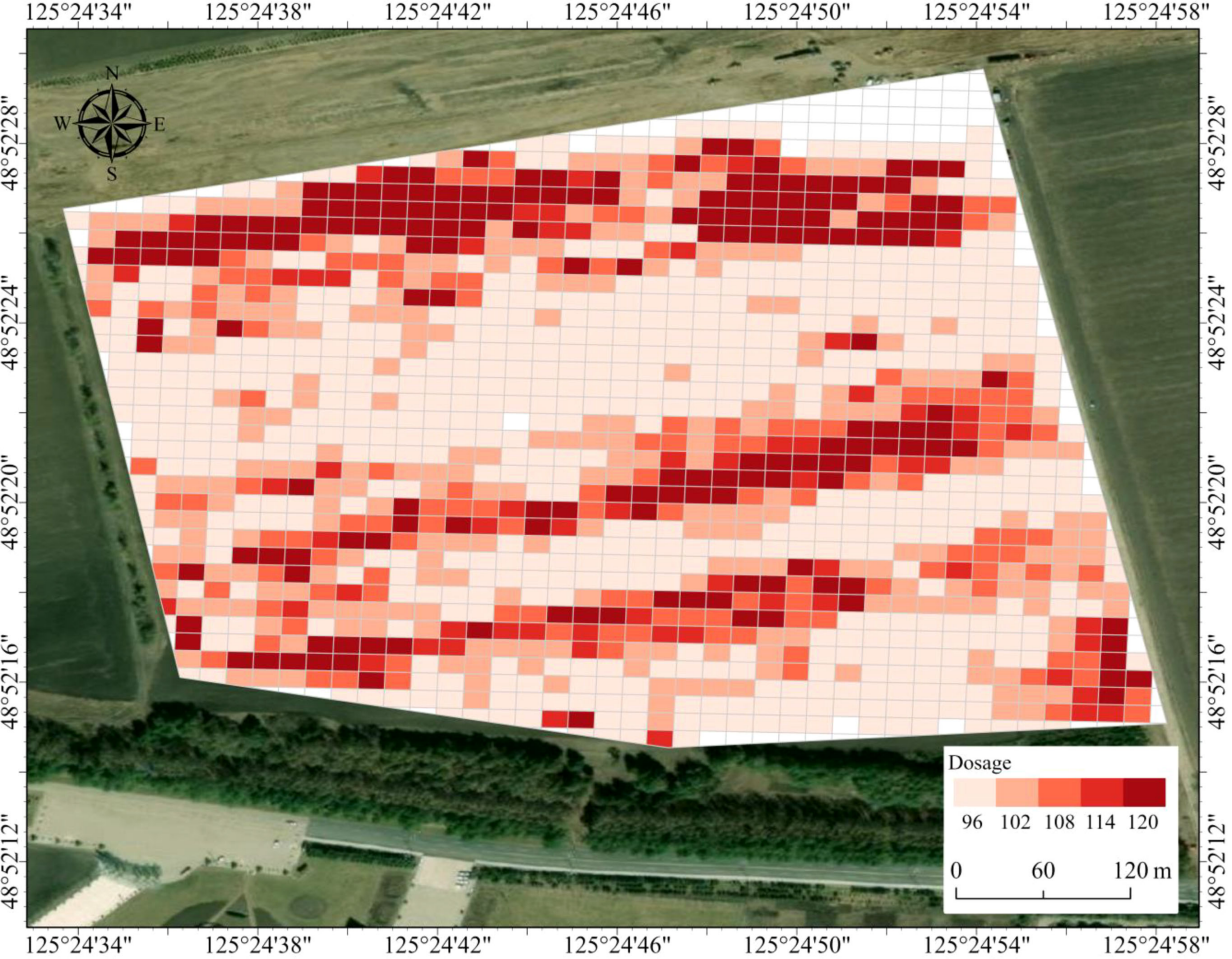
Large-scale variable-rate herbicide application map.

Leveraging CPWC and UAV-based weed spatial data, this study designed a variable-rate herbicide prescription map for the true leaf stage. The design was based on a decision rule that maps the five weed density levels to corresponding application rates. Using a locally recommended baseline rate of 120 L/hm² for conventional blanket application at this growth stage, application rates were adjusted downward in 5% increments for each lower density level, culminating in a 20% reduction for the lowest-density grids. This formed five application levels: 120, 114, 108, 102, and 96 L/hm², corresponding to density levels from highest to lowest. The 5% adjustment interval was set to ensure operational practicality while providing a meaningful gradient in chemical reduction. This spatially differentiated approach optimizes herbicide use and supports precision spraying decisions, with the specific density-to-rate mapping visualized in the prescription map (as shown in **Figure 15**).

## 4 Discussion

This study proposes a method for precise weed segmentation and variable-rate herbicide application map generation based on an improved YOLOv11-seg framework, aimed at monitoring soybean field weeds during the seedling stage. The results demonstrate that this approach significantly enhances weed recognition accuracy in complex field environments and provides reliable spatial information support for precision agriculture.

Ablation experiments verified the effectiveness of each module. The RCSOSA module, through multi-scale receptive field fusion, enhances the feature extraction capability for small and densely distributed weeds. The SEAM attention mechanism strengthens both spatial and channel feature representations, markedly improving recognition performance under occlusion and morphologically similar conditions. The iRMB structure improves feature fusion in the detection head, while the ADown dual-branch downsampling module effectively preserves low-contrast fine features. The synergistic effect of these modules achieves higher precision and recall while maintaining relatively low computational complexity, with overall performance surpassing that of YOLOv8s-seg, YOLOv11s-seg, and YOLOv12s-seg.

Low-altitude UAV remote sensing provides high-resolution imagery, accurately reflecting the spatial distribution of crops and weeds, and offers flexible and rapid data acquisition. However, factors such as illumination changes, shadow interference, and plant occlusion may still affect detection results. Although the improved model demonstrates strong robustness, future work could integrate multi-temporal, multi-angle, and multispectral imagery to further enhance generalization performance.

Weed density maps generated from the segmentation results intuitively display the spatial heterogeneity of weeds in the field. By dividing the field into 12 m × 12 m grids corresponding to the width of ground sprayers, variable-rate herbicide application areas can be automatically delineated. Minor deviations in weed coordinates due to RTK positioning accuracy and image resolution have a limited impact on overall spraying planning. The natural breaks method performs well in classifying weed density, though future studies could explore adaptive classification strategies based on machine learning to further optimize herbicide application.

Regarding the choice of evaluation metrics, although COCO-style mAP@ [0.5:0.95], AP at different scales (small, medium, large), and the mean ± standard deviation from multiple training runs provide a more comprehensive assessment of instance segmentation performance, the primary targets in this study are early-stage, extremely small weeds. Existing research on UAV-based weed detection predominantly uses mAP@0.5 as the key metric, as high IoU thresholds often lead to unstable or uninterpretable evaluations in scenarios involving very small targets. Moreover, training segmentation models on high-resolution UAV imagery is computationally intensive, and conducting multiple complete training cycles demands significant computational resources and time. Consequently, this study employs mAP@0.5 as the core evaluation metric. Future work, given sufficient computational resources, will incorporate COCO-style AP metrics and repeated experiments to enhance the completeness and rigor of the evaluation framework.

This method exhibits strong scalability and practical potential. Its lightweight network architecture is suitable for real-time deployment on UAVs or edge devices, and when combined with GIS spatial analysis, enables a complete workflow from image acquisition and weed recognition to herbicide application decision-making, providing a feasible technical pathway for intelligent weed management. Future research will focus on multi-source data fusion, temporal dynamic monitoring, and integration with autonomous spraying systems to achieve closed-loop precision agriculture management.

## 5 Conclusion

Addressing the challenges of low accuracy in weed recognition during the soybean seedling stage, complex backgrounds, and severe target occlusion, this study proposes a task-driven model integration improvement scheme and a corresponding engineering decision-making workflow to meet the practical requirements of precision weeding. A high-precision weed segmentation and spatial decision-making method was developed based on an improved YOLOv11-seg. By strategically integrating the RCSOSA, SEAM, iRMB, and ADown modules, the model significantly enhances multi-scale feature extraction and attention aggregation capabilities. This effectively improves the robustness in identifying dense and small-target weeds, achieves real-time inference while maintaining a lightweight architecture, and demonstrates feasibility for field applications.

Experimental results show that the improved YOLOv11-seg outperforms classic segmentation models such as Mask R-CNN and YOLOv8s-seg in terms of precision, recall, and mAP. Ablation studies confirm the synergistic effects of the integrated modules in multi-scale feature fusion, attention optimization, and low-level feature preservation, leading to significantly improved detection performance in complex scenarios. A complete technical pathway from perception to decision-making was established. Leveraging UAV imagery and GIS spatial analysis, a weed density map and a variable-rate spraying decision framework were generated, providing an actionable, spatialized solution for precision weeding and forming a closed-loop technical path from perceptual recognition to agronomic decision-making.

In summary, through task-oriented model integration and end-to-end engineering implementation, the results of this study fully validate the feasibility and potential for broader application of combining deep learning with UAV remote sensing in intelligent weed management. The lightweight design of the model is suitable for real-time deployment on UAVs or edge computing devices, providing efficient technical support for large-scale precision agriculture. Future work will focus on expanding to multi-region, multi-crop, and multi-temporal datasets, further enhancing model generalization by incorporating multispectral and temporal analysis, and linking with autonomous variable-rate application systems to realize an intelligent, closed-loop weed control system. The proposed high-precision weed segmentation and spatial decision-making method not only achieves deep integration of model architecture and spatial application but also provides a significant technical reference for the precision, green development, and sustainability of smart agriculture.

## Data Availability

The original contributions presented in the study are included in the article/supplementary material, Further inquiries can be directed to the corresponding author.
